# Identification and pathogenicity analysis of leaf brown spot of *Juglans regia* in China

**DOI:** 10.1038/s41598-023-33853-1

**Published:** 2023-04-22

**Authors:** Feihu Wang, Chao Liu, Qian Zeng, Yijie Zhou, Feng Liu, Xiulan Xu, Hanbo Yang, Yinggao Liu, Chunlin Yang

**Affiliations:** 1 Yangtze River Upper Reaches Forest Resources Conservation and Ecological Safety Key Laboratory of the National Forestry and Grassland Administration & Research Institute of Forestry in the Upper Reaches of the Yangtze River, College of Forestry, Sichuan Agricultural University, Chengdu, 611130 Sichuan China; 2 Chengdu Academy of Agricultural and Forestry Sciences, Forestry Research Institute, Chengdu, 611130 Sichuan China

**Keywords:** Microbiology, Industrial microbiology, Pathogens

## Abstract

English walnut (*Juglans regia*), has high economic and ecological value. As an important tree species for eliminating poverty, it is planted in many Provinces of China. In 2021, new pathogenic fungi were observed in English walnut in Guangyuan City, Sichuan Province, China. The initial symptom of leaf infection is that the leaves are covered with small black spots, which gradually expand into larger brown spots. Most of the spots appeared at the edges of the leaves, and yellow whorls were observed at the junction between the spots and the healthy leaves. The pathogenic fungi were isoalted form collecting disease samples and purified by single-spore culturing. In vitro and field experiments showed that the pathogen could cause brown spots on walnut leaves. The inoculation experiment showed that the symptoms in the field experiment were the same as those observed on the spot; however, slight differences were observed in the in vitro experiment. Ten isolates were obtained from walnut leaves with brown spot symptoms, and these were further characterized based on morphology and DNA sequencing. ITS (internal transcribed spacer), LSU (large sub-unit rDNA), *rpb*2 (second largest subunit of RNA polymerase) and *tub*2 (beta-tubulin) gene regions were used to construct phylogenetic trees and determine the evolutionary relationships among the collected strains. The isolate was identified as *Nothophoma quercina* by morphological and polygene analyses. As far as we are aware, the brown spots on walnut leaves caused by *N. quercina* is the first report of its kind.

## Introduction

English walnut (*Juglans regia*), belonging to the family Juglandaceae, is a nut tree species whose seeds have high nutritional and economic value and are widely distributed globally^1^. The *J. regia* cultivation has a long history in China. This tree species also has a high economic value. The seeds can be used to squeeze extract oil. English walnuts are also becoming increasingly popular due to their high edible value, economic benefits, and adaptability to different soils and climates. With the rapid development of economic forests, walnut planting has become popular in counties, cities, and districts of China. Walnut is the main cash crop in some areas and accounts for approximately 80% of the local income. In 2020, approximately 600, 000 tons of shell walnut seeds were produced in the Sichuan Province. In China, the output and planting area rank third and second, respectively. Owing to the continuous increase in the planting area, the same type of cultivation methods is used under different site conditions and the varieties are frequently introduced from outside the region. Previously rare diseases in this area have become increasingly serious. Different diseases such as anthracnose, branch blight, and walnut rot appear consecutively. In this study, we found that a new disease was emerging. In 2021, walnut leaves with brown spots were found in a walnut garden in Guangyuan City (Sichuan Province, China). Symptoms included a large number of tan spots around the leaves, a black protrusion in the center, and a yellow halo at the junction of the affected part and the healthy leaves. In the late stage, the infected leaves were covered with small pycnidia and turned from dark brown to black, leading to early defoliation. In 2021, nearly 40% of the 50 walnut trees investigated exhibited the same symptoms, causing significant yield loss. Although the yield loss of walnut caused by this pathogen has not been reported yet, the disease causes considerable yield losses in many cherries growing regions^2^. In recent years, research on walnuts has mainly focused on the optimization of germplasm resources, and approximately 80 cultivars have been generated so far around in Sichuan Province. However, there are few reports on walnut diseases, which are not conducive to the management and operation of the walnut industry in the Sichuan province.

*Nothophoma* was described by Chen et al.^3^ There are currently 22 species in this genus. Many species in this genus are plant pathogens, and its members cause diseases in a wide variety of woody plants^3–5^. Several important fruits, including *Chaenomeles sinensis*, have been reported to be affected by *Nothophoma* genus fungi in Korea. *Nothophoma quercina* species are important plant pathogens, have a wide range of economically important plant hosts. The disease symptoms in fruit and nut trees include brown spot of jujube, *Aucuba japonica* leaf blight, bud blight on *Photinia* × *fraseri*, leaf blight of *Magnolia coco*, leaf spot disease of *Phellodendron amurense* and trunk canker on *Malus micromalus*^6–11^. All of these cases were induced by *N. quercina*. These results confirmed their pathogenicity on hosts as symptoms were reproduced. There have, however, been no reports of walnut brown spots caused by *N. quercina.* This study aimed to identify the pathogen responsible for walnut brown spot in Sichuan Province, China.

## Materials and methods

### Sample collection and isolation

The occurrence of walnut brown spot in walnut main producing area of Guangyuan City, Sichuan Province, China was investigated. Guangyuan is located in the northeast of Sichuan Province (36 32′ n, 105 52′E). Located in the south of the Qinling Mountains, this area is the north–south boundary zone of China, and the climate is a typical subtropical humid monsoon climate. The recorded annual average temperature is 16.1 °C and the annual rainfall is 800 to 1000 mm. The main walnut varieties sampled from 6 orchards were “Shuoxing”, “Xiazao”, “Chuanzao 2”, and “Shuchao 2”, with an average orchard age of 14 years. Samples were collected in spring and summer from 2021 to 2022. Fifteen walnut trees of the same age were selected from each orchard. Samples include leaves with obvious disease spots and dead leaves. Plastic Ziploc bags were used to transport samples to the laboratory. A procedure described by Wijesinghe was followed to examine and process the samples^12^. An NVT-GG dissecting microscope was used to observe and photograph pycnidia external shape, size, and color (Company for cutting-edge photoelectricity technology in Shanghai) VS-800C micro-digital camera matched (Weishen Times Technology Co., Ltd., Shenzhen, China). The microstructure of conidiophores and conidia was observed and photographed using an OLYMPUS BX43 compound microscope and an OLYMPUS DP22 digital camera. Tarosoft® Image Frame Work v.0.9.7 was used to observe and measure the following structures: diameter, height, color, and shape of Conidiophores; length and width of Conidia (Measure the maximum and minimum values and determine the range). Some images are processed by Adobe Photoshop CS6. And describe according to the morphological characteristics. Before separation, wash the blade surface with sterile distilled water for 3 times to remove surface dust. Surface-sterilized in 75% ethanol for 30 s, washed three times with sterile water, and dried at room temperature. Single spore was isolated by microscope and cultured in PDA plate. According to Chomnunti method, pure cultures were obtained from single conidia on potato-dextrose agar (PDA) media^13^. Cultures were incubated at 25 °C for up to 1 month. Colony characteristics were initially used to identify fungal isolates obtained from walnuts (density, texture, pattern, rate and color of mycelial growth, and presence or absence of pycnidia) and conidial morphology (shape, color, absence or presence of septation of conidia) into *Nothophoma* genera. The type specimens collected in the field are kept in the Herbarium of Sichuan Agricultural University, Chengdu, China (SICAU). The strains are deposited in the fungal specimen preservation room of Sichuan Agricultural University (SICAUCC).

### DNA extraction and polymerase chain reaction (PCR) amplification

DNA was extracted from fresh mycelia with a DNA extraction kit™ (TIANDZ, China), following the specifications of the kit. Operate and use according to the instruction of the reagent. The extracted DNA is stored at 4 °C for normal use, and the long-term storage should be kept at − 20 °C. The primers LR0R and LR7, ITS5 and ITS4, fRPB2-5F and fRPB2-7cR and Bt2a and Bt2b^14–17^. Were used for the amplification of the internal transcribed spacers (ITS), large sub-unit rDNA (LSU), the second largest subunit of RNA polymerase II (*rpb*2) and β-tubulin (*tub*2), respectively. Polymerase chain reaction (PCR) amplification was carried out following Dai et al.^18^.

The PCR reaction system was 25 μl, comprises mainly 22 μl of Jingpai PCR mixture (TSINGKE, Chengdu, China), add 1 μl of upstream primer and 1 μl of downstream primer, at last, 1 μl template genomic DNA was added. PCR execution procedure is as follows: LSU, and ITS have the same running procedures. Firstly, denature at 94 °C for 3 min; then denature at 94 °C for 30 s for 35 cycles, annealing at 55 °C for 50 s and elongation at 72 °C for 60 s. Finally, it was extended at 72 °C for 10 min. The following procedures are adopted for *rpb2*: Firstly, denature at 95 °C for 5 min; then denature at 95 °C for 1 min for 35 cycles, annealing at 52 °C for 2 min and elongation at 72 °C for 90 s; finally, it was extended at 72 °C for 10 min. The following procedures are adopted for *tub*2: firstly, denature at 94 °C for 3 min; then denature at 94 °C for 3 s for 35 cycles, annealing at 52 °C for 50 s and elongation at 72 °C for 1 min; finally, it was extended at 72 °C for 10 min. The TSINGKE (Chengdu, China) Biological Technology Co., Ltd, was contracted to sequence successfully amplified PCR products. The consensus sequences were obtained from generated sequence reads using Bioedit version 7.0.5.3 software. All the obtained sequences were deposited in NCBI GenBank.

### Phylogenetic analysis

The sequences of closely related strains were determined by BLAST search in GenBank. We confirmed and obtained all molecular data of *Nothophoma* following recent studies^8,19^. The sequences were downloaded from GenBank. (http://www.ncbi.nlm.nih.gov/). Single gene and phyloygene (LSU, ITS, *rpb2* and *tub*2) Compare all sequences MAFFT v.7 (https://mafft.cbrc.jp/alignment/server/)^20^. If necessary, use BioEdit v.7.0.5.2 software to make manual improvement^21^. Manually delete the aligned front and back ends and blurred areas. By comparing the single gene sequence data, the consistency is checked, and the overall topological structure is determined. Polygene sequence consists of Mesquite version 3.11 (build 766)^22^. Combining the Bayesian inference (BI) and maximum likelihood analysis (ML) analysis, the phylogenetic analysis of ITS, LSU, *rpb*2 and tub2 gene sequences was carried out. Use Clustal X version 1.81 to convert alignment to NEXUS file (.nxs)^23^, then BI analysis is carried out. According to the Akaike Information Criterion (AIC), MrModeltest. v.2.2 determined the best nucleotide substitution model^24^. Maximum likelihood analysis using CIPRES science gateway network server^25^, and chosen RAxML-HPC2 on XSEDE (8.2.10)^26^ GTRGAMMA substitution model is used for 1000 boot iterations. Bayesian analysis was performed by MR Bayes v.3.2.2^27^. Run 6 Markov chains at the same time, totaling 5000 generations, and sample the tree every 100 generations. The aging frequency is set to 0.25, and when the average standard deviation of splitting frequency reaches below 0.01, the system will automatically stop running^28^. Phylogenetic tree was visualized by Fig Tree v.1.4.3^29^. In addition, Adoble Illustrator CS6 v.16.0.0 is used for layout and improvement. The maximum likelihood guidance value (MLBP) is equal to or greater than 63%. Bayesian posterior bootstrap values (BYPP) is greater than 0.54. All sequences used and generated in this paper are submitted to GenBank and listed in Table 1.

**Table 1:** Taxa used in the phylogenetic analyses and their corresponding GenBank numbers.

Species	Strain numbers	Hosts	Countries	ITS	TUB	LSU	RPB2
*Allophoma labilis*	CBS 124.93	*Lycopersicon esculentum*	Netherlands	GU237765	GU237619	GU238091	KT389552
*Allophoma minor*	CBS 325.82	*Syzygium aromaticum*	Indonesia	GU237831	GU237632	GU238107	KT389553
*Allophoma nicaraguensis*	CBS 506.91	*Coffea arabica*	Nicaragua	GU237876	GU237596	GU238058	KT389551
*Allophoma tropica*	CBS 436.75	*Saintpaulia ionantha*	Germany	GU237864	GU237663	GU238149	KT389556
*Ascochyta boeremae*	CBS 372.84	*Pisum sativum*	Australia	KT389480	KT389774	KT389697	NA
*Ascochyta phacae*	CBS 184.55	*Phaca alpina*	Switzerland	KT389475	KT389769	KT389692	NA
*Ascochyta rabiel*	CBS 534.65	*Cicer arietinum*	India	GU237886	GU237533	GU237970	KP330405
*Boeremia diversispora*	CBS 102.80	*Phaseolus vulgaris*	Kenya	GU237725	GU237492	GU237930	KT389565
*Boeremia foveata*	CBS 109176	*Solanum tuberosum*	Bulgaria	GU237742	GU237508	GU237946	KT389578
*Boeremia noackiana*	CBS 100353	*Phaseolus vulgaris*	Guatemala	GU237710	GU237514	GU237952	NA
*Briansuttonomyces eucalypti*	CBS 114879	*Eucalyptus* sp.	South Africa	KU728479	KU728595	KU728519	NA
*Calophoma clematidina*	CBS 108.79	*Clematis* sp.	Netherlands	MH861182	FJ427100	FJ515632	KT389588
*Calophoma complanata*	CBS 100311	*Heracleum sphondylium*	Netherlands	GU237709	GU237594	EU754181	KT389590
*Calophoma vodaki*	CBS 173.53	*Hepatica triloba*	Switzerland	KT389497	KT389791	KT389714	NA
*Didymella americana*	CBS 185.85	*Zea mays*	USA	FJ426972	FJ427088	GU237990	KT389594
*Didymella anserina*	CBS 285.29	*Calluna* sp.	UK	KT389499	KT389796	KT389716	NA
*Didymella mascrostoma*	CBS 223.69	*Acer pseudoplatanus*	Switzerland	GU237801	GU237623	GU238096	KT389608
*Didymella sancta*	CBS 281.83	*Ailanthus altissima*	South Africa	FJ427063	FJ427170	GU238030	KT389623
*Epicoccum brasiliense*	CBS 120105	*Amaranthus* sp.	Brazil	GU237760	GU237588	GU238049	KT389627
*Epicoccum draconis*	CBS 186.83	*Dracaena* sp.	Rwanda	GU237795	GU237607	GU238070	KT389628
*Epicoccum nigrum*	CBS 125.82	Human toenail	Netherlands	FJ426995	FJ427106	GU237974	KT389631
*Epicoccum sorghinum*	CBS 179.80	*Sorghum vulgare*	Puerto Rico	FJ427067	FJ427173	GU237978	KT389635
*Heterophoma adonidis*	CBS 114309	*Adonis vernalis*	Sweden	KT389506	KT389803	KT389724	KT389637
*Heterophoma poolensis*	CBS 116.93	*Antirrhinum majus*	Netherlands	GU237755	GU237649	GU238134	NA
*Heterophoma sylvatica*	CBS 874.97	*Melampyrum pratense*	Netherlands	GU237907	GU237662	GU238148	NA
*Leptosphaerulina arachidicola*	CBS 275.59	*Arachis hypogaea*	Taiwan China	GU237820	GU237543	GU237983	NA
*Leptosphaerulina australis*	CBS 317.83	*Eugenia aromatica*	Indonesia	GU237829	GU237540	EU754166	GU371790
*Leptosphaerulina trifolii*	CBS 235.58	*Trifolium* sp.	Netherlands	GU237806	GU237542	GU237982	NA
*Macroventuria anomochaeta*	CBS 502.72	*Medicago sativa*	South Africa	GU237873	GU237545	GU237985	NA
*Neoascochyta argentina*	CBS 112524	*Triticum aestivum*	Argentina	KT389524	KT389822	KT389742	NA
*Neoascochyta graminicola*	CBS 301.69	*Lolium multiflorum*	Germany	KT389519	KT389817	KT389737	KT389650
*Neoascochyta triticicola*	CBS 544.74	*Triticum aestivum*	South Africa	GU237887	GU237488	EU754134	KT389652
*Neodidymelliopsis cannabis*	CBS 121.75	*Urtica dioica*	Netherlands	GU237761	GU237535	GU237972	NA
*Neodidymelliopsis Polemonii*	CBS 375.67	*Polemonium caeruleum*	Netherlands	KT389530	KT389828	KT389748	NA
*Neodidymelliopsis xanthina*	CBS 168.70	*Delphinium* sp.	Netherlands	KT389533	KT389831	KT389751	NA
*Nothophoma acaciae*	CBS 143404	*Acacia melanoxylon*	Australia	MG386056	MG386167	MG386109	MG386144
*Nothophoma anigozanthi*	CBS 381.91	*Anigozanthus maugleisii*	Netherlands	GU237852	GU237580	GU238039	KT389655
*Nothophoma arachidis-hypogaeae*	CBS 125.93	*Arachis hypogaea*	India	GU237771	GU237583	GU238043	KT389656
*Nothophoma brennandiae*	CBS 140541	House dust	Canada	MN973558	MT005661	MN943765	MT018202
*Nothophoma chromolaenae*	MFLUCC 17-1443	*Chromolaena odorata*	Thailand	MT214364	NA	MT214458	NA
*Nothophoma eucalyptigena*	CBS 142535	*Eucalyptus*	Australia	KY979771	KY979935	KY979826	KY979852
*Nothophoma garlbiwalawarda*	BRIP 69595	*Senna artemisioides*	Australia	MN567686	NA	NA	MN604937
*Nothophoma infuscata*	CBS 121931	*Acacia longifolia*	New Zealand	MN973559	MT005662	MN943766	MT018203
*Nothophoma quercina*	CBS 633.92	*Quercus* sp.	Ukraine	GU237900	GU237609	EU754127	KT389657
***Nothophoma quercina***	**SICAUCC 22-0080**	***Juglans regia***	**China**	**ON707468**	**ON645232**	**ON707463**	**ON645227**
***Nothophoma quercina***	**SICAUCC 22-0081**	***Juglans regia***	**China**	**ON707469**	**ON645233**	**ON707464**	**ON645228**
***Nothophoma quercina***	**SICAUCC 22-0082**	***Juglans regia***	**China**	**ON707470**	**ON645234**	**ON707465**	**ON645229**
***Nothophoma quercina***	**SICAUCC 22-0083**	***Juglans regia***	**China**	**ON707471**	**ON645235**	**ON707466**	**ON645230**
*Nothophoma gossypiicola*	CBS 377.6	*Gossypium* sp.	USA	GU237845	GU237611	GU238079	KT389658
*Nothophoma gossypiicola*	CBS 377.6	*Gossypium* sp.	USA	GU237845	GU237611	GU238079	KT389658
*Nothophoma infossa*	CBS 123395	*Fraxinus pennsylvanica*	Argentina	FJ427025	FJ427135	GU238089	KT389659
*Nothophoma macrospora*	CBS 140674	Human	USA	LN880536	LN880539	LN880537	LT593073
*Nothophoma naiawu*	BRIP 69583	*Senna artemisioides*	Australia	MN5676787	NA	NA	MN5676787
*Nothophoma ngayawang*	BRIP 69582	*Senna artemisioides*	Australia	MN567688	NA	NA	MN604939
*Nothophoma nullicana*	CPC 32330	*Acacia falciformis*	Australia	NR 156,665	NA	NA	MH853662
*Nothophoma prosopidis*	CPC 21699	*Prosopis* sp.	South Africa	KF777149	NA	KF777204	NA
*Nothophoma pruni*	MFLUCC 18-1601	*Malus micromalus*	China	MH827005	MH853669	MH827026	MH853662
*Nothophoma pruni*	JZB 380015	*Prunus avium*	China	MH827004	MH853668	MH827025	MH853661
*Nothophoma raii*	A 189	Soil	India	MF664467	MF664468	NA	NA
*Nothophoma spiraeae*	CFCC 53928	*Spiraea salicifolia*	China	MN737833	MN879295	MN737828	MN879292
*Nothophoma spiraeae*	CFCC 53929	*Spiraea salicifolia*	China	MN737834	MN879296	MN737829	MN879293
*Nothophoma spiraeae*	CFCC 53930	*Spiraea salicifolia*	China	MN737832	MN879297	MN737830	MN879294
*Nothophoma variabilis*	DI16-285	Human	USA	LT592939	LT593008	LN907428	LT593078
*Paraboeremia adianticola*	CBS 187.83	*Polystichum adiantiforme*	USA	GU237796	GU237576	GU238035	KP330401
*Paraboeremia putaminum*	CBS 372.91	*Ulmus* sp.	Netherlands	GU237843	GU237651	GU238137	NA
*Paraboeremia selaginellae*	CBS 122.93	*Selaginella* sp.	Netherlands	GU237762	GU237656	GU238142	NA
*Phoma herbarum*	CBS 304.51	*Achillea millefolium*	Switzerland	KT389538	KT389836	KT389755	NA
*Phomatodes aubrietiae*	CBS 383.67	*Aubrietia hybrida*	Netherlands	GU237854	GU237584	GU238044	NA
*Phomatodes nebulosa*	CBS 117.93	*Mercurialis perennis*	Netherlands	GU237757	GU237633	GU238114	KP330425
*Stagonosporopsis actaeae*	CBS 114303	*Actaea spicata*	Sweden	KT389544	KT389847	KT389760	NA
*Stagonosporopsis helianthi*	CBS 200.87	*Helianthus annuus*	Italy	KT389545	KT389848	KT389761	KT389683
*Xenodidymella applanata*	CBS 195.36	*Rubus idaeus*	Netherlands	KT389548	KT389852	KT389764	NA
*Xenodidymella catariae*	CBS 102635	*Nepeta catenaria*	Netherlands	GU237727	GU237524	GU237962	KP330404
*Xenodidymella humicola*	CBS 220.85	*Franseria* sp.	USA	GU237800	GU237617	GU238086	KP330422

The newly uploaded gene sequence is displayed in bold black.

### Pathogenicity tests

Four isolates were selected for the subsequent pathogenicity test. Before inoculation, the isolates were cultured on Potato Dextrose Agar (PDA) at 25 °C for 15 days under 12 h fluorescent light/dark conditions.

### Pathogenicity on walnut plant

The pathogenicity test was performed on six plant 2 year-old potted walnut. The 2-year-old grafted seedlings were collected and cultured in the greenhouse. The spore suspension was then sprayed onto the tender leaves of cuttings from the three seedlings. Sterile water was sprayed on the tender leaves of three grafted seedlings as a control. All six treatments were bagged and the relative humidity was ≥ 90%. The seedlings were kept in a greenhouse for 30 days. Then, the collected healthy young leaves were disinfected in 3% sodium hypochlorite for 60 s and 75% ethanol for 60 s, washed in sterile water 3–5 times, dried in air, and finally inoculated with conidia suspension (4.5 × 10^5^ conidia/mL). During the experiment, 12 healthy young leaves were sprayed with spore suspension, and the remaining 12 leaves were sprayed with sterile water. The inoculated leaves were placed on a flat plate sterilized at high temperature and placed in a constant temperature light incubator at 25 °C (relative humidity 90%) for cultivation and observation. Every 7 day after inoculation, the disease severity was evaluated by the percentage (dpi) by measuring the leaf surface size with disease symptoms. This experiment was repeated three times.

### Ethics statement

All plant materials and fungal specimens are treated according to international standards.

## Results

### Disease symptoms identification

**Figure 1:**
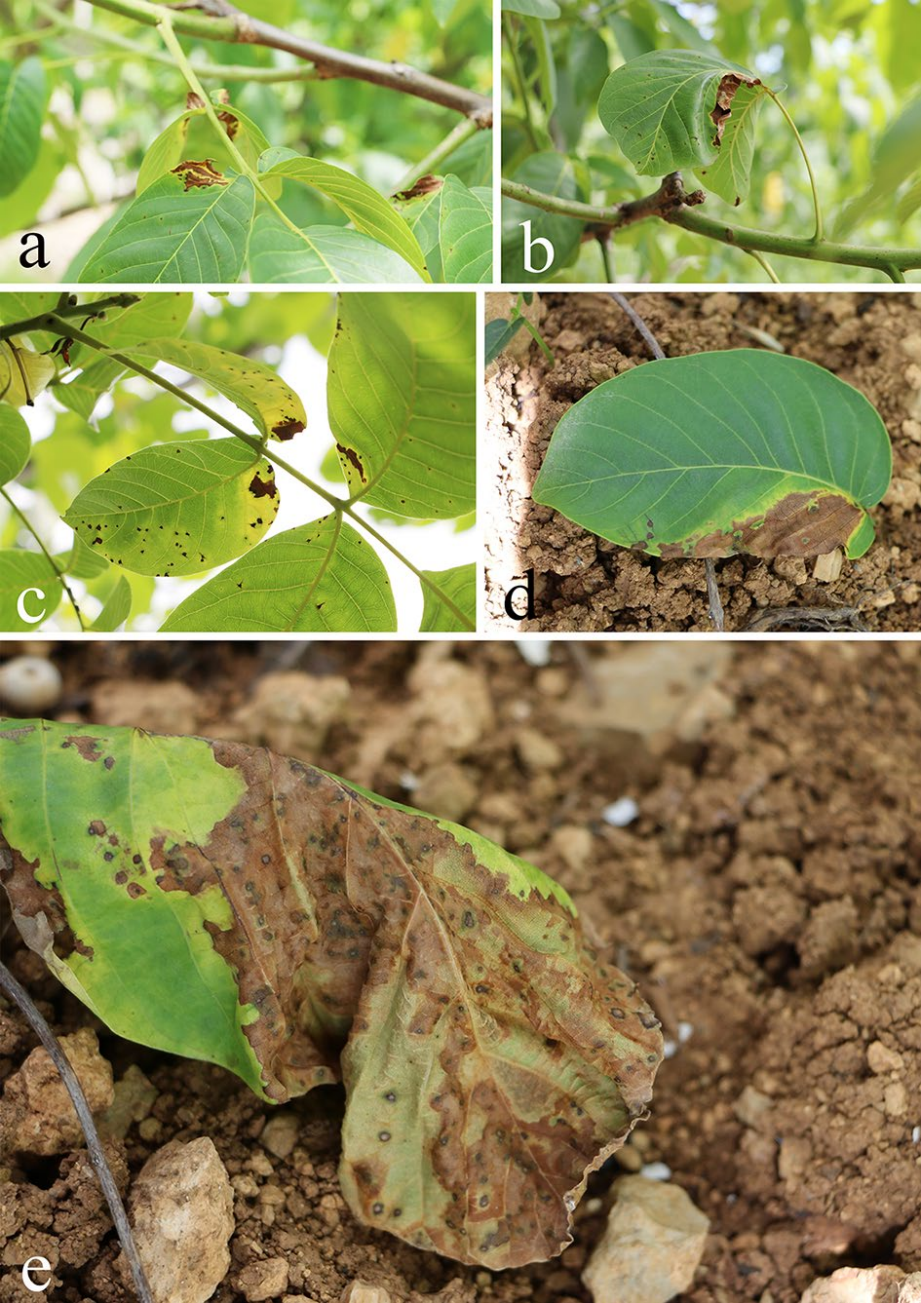
Disease symptoms of leaves on *Juglans regia* in the field. (**a**–**c**) Leaf brown necrosis symptoms; (**d**), Local leaf blight due to enlargement of the spot; (**e**) Leaf withered and fallen with severe infection.

With typical brown necrotic spots (Fig. 1a) a walnut orchard with typical disease spots was observed in the Chaotian District of Guangyuan City (36°32′28″N, 105°52′26″E, 557 m above sea level), Sichuan Province, China. All symptoms were observed in all the six orchards surveyed. These small brown necrotic areas appeared on new leaves, first from the tip and edge of the leaf, and developed inward. The lesion expanded to form a dark brown necrotic area and some leaves had brownish-red edges (Fig. 1a,c). This leads to the distortion and necrosis of the leaves (Fig. 1b). Finally, the leaves died and fell in advance (Fig. 1d,e). In June 2020, a large number of typical symptoms of walnut brown spot were found in the walnut base in Guangyuan City, Sichuan Province. the total planting area of walnut in this base is about 1000 hectares. Ninety samples with typical symptoms were collected from six orchards, and the fungi isolated from the 60 samples were morphologically similar to *Nothophoma quercina*. According to the statistical analysis of collected samples, the incidence of walnut leaf brown spot is about 40%.

### Isolation of fungi

**Figure 2:**
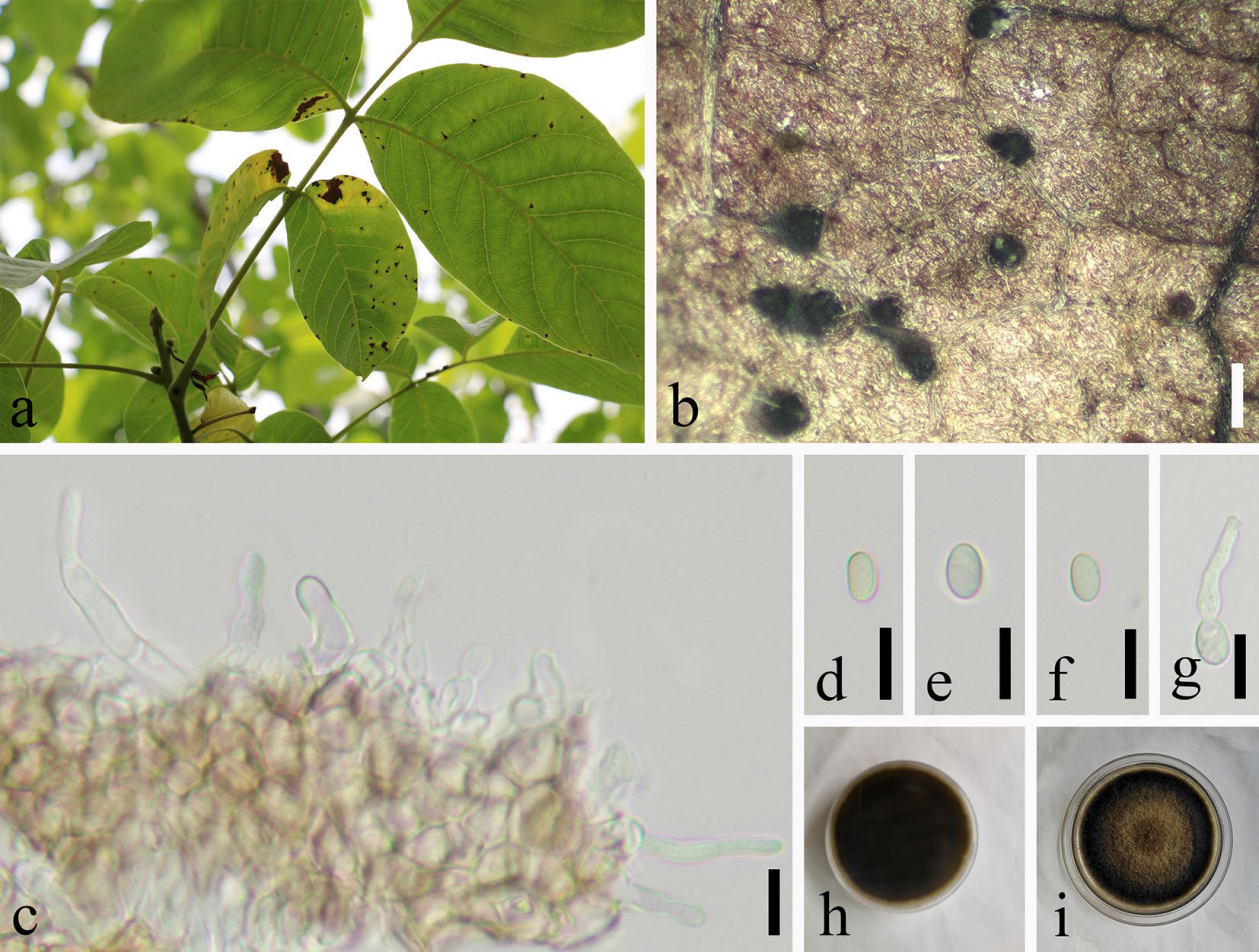
Phenotypic traits of *Nothophoma quercina*. (**a**) symptoms caused by *N. quercina*; (**b**) Conidiomata on leaf surface; (**c**) Conidiogenous cells with developing conidia; (**d**–**f**) Conidia; (**g**) Germinating conidium; (**h**,**i**) Cultures on PDA. Scale bars: (**b**) = 500 μm; (**c**–**g**) = 10 μm.

Ten isolates were isolated from diseased leaves with walnut brown spots (Fig. 2a), which were cultured on PDA medium using the same method as Chomnunti et al.^13^ Conidia germinate in 24 h (Fig. 2g). colonies grow in a circle, with regular edges and wool-like hypha; the aerial hyphae are white at first, aerial hyphae were initially white, after the seventh day, it gradually changed from brown to dark brown at 25 °C under a 12 h fluorescent light/dark regime. The colonies are distributed in a circular cushion shape, and the aerial hyphae are lighter in color (Fig. 2h,i). After 14 ~ 20 days of culture, a large number of dark brown pycnidia were observed on the surface of the culture medium. Pycnidia were measurement (210–)220–360(− 365) × 190(− 195)–(230–)240 μm (${\overline{\text{x}}}$ ± SD = 290 ± 4 × 205 ± 3 μm, n = 20), drown to dark brown, hemispherical, spherical or irregular. Conidia measurement size (3.7–)4.2–8 × 2.7(– 3)–(4.8–)5.6 μm (${\overline{\text{x}}}$ ± SD = 6.1 ± 0.6 μm × 4.1 ± 0.4 μm, n = 50), most of them are oval, partially circular, smooth, hyaline, aseptate, thick-walled, Light brown at maturity (Fig. 2b–f). Isolates with small morphological differences were then compared with isolates from different hosts with larger conidia and pycnidia. This morphological similarity was higher than that of the type species. It was found that there was no significant difference in the size of conidia among the isolates. However, the differences of pycnidial size among isolates were obvious. The host plants of *Nothophoma quercina* from different locations differ, however, no reports have been found on walnut plants.

### Phylogenetic analysis

**Figure 3:**
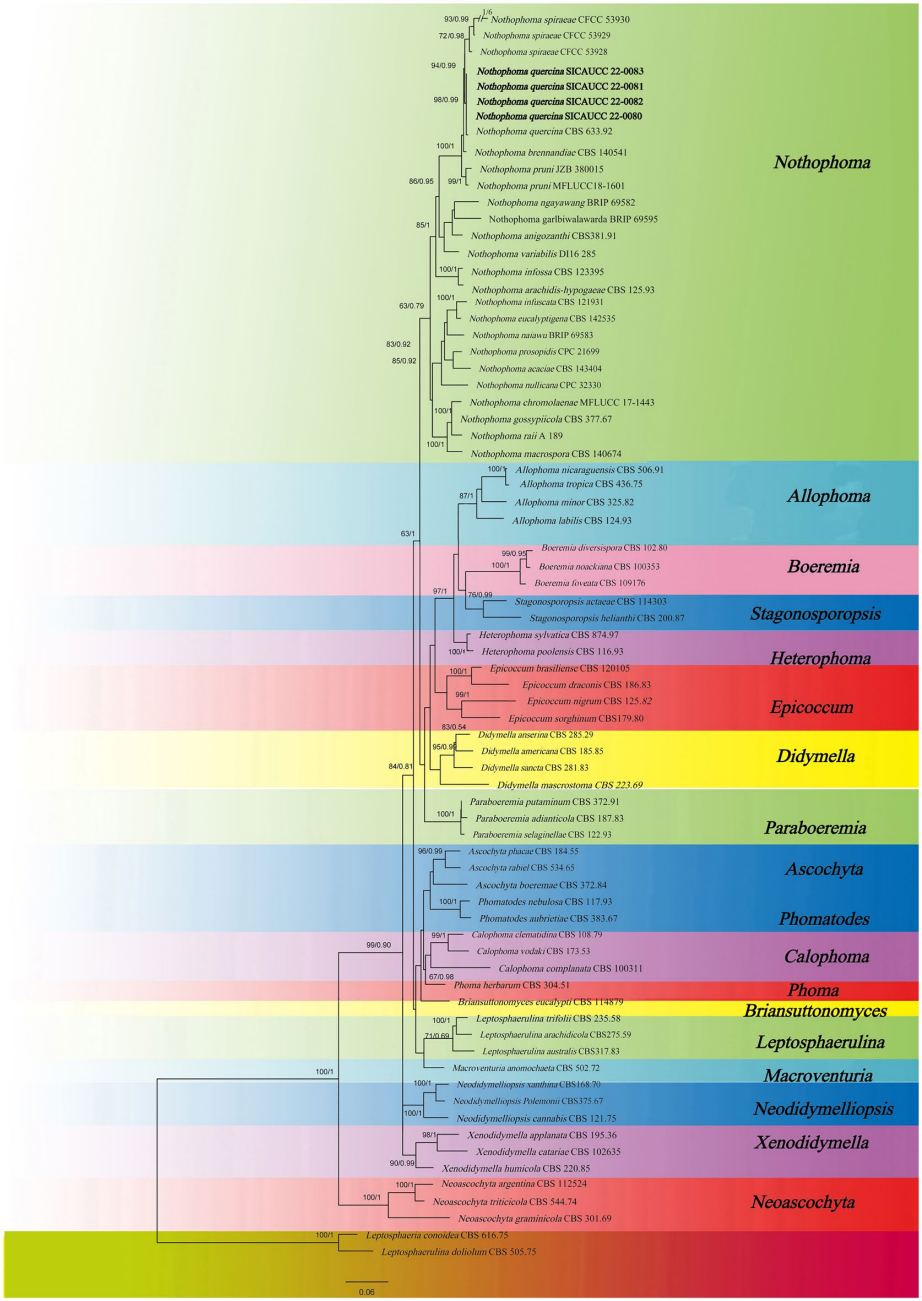
RAxML tree based on a combined dataset of a partial LSU, ITS, *tub*2 and *rpb*2 sequence analysis in Didymellaceae. The tree is rooted with *Leptosphaeria conoidea* (CBS 616.75) and *L. doliolum* (CBS 505.75). ML and Bayesian posterior probabilities values are given at the nodes. The new isolate is highlighted in bold.

Only morphological identification can no longer meet the needs of current identification. In order to verify the accuracy of our morphological identification, we also added molecular biological identification. So as to determine its pathogen. Gene fragments of LSU, ITS, *tub*2 and *rpb*2 in *Nothophoma quercina* (SICAUCC 22-0080, SICAUCC 22-0081, SICAUCC 22-0082 and SICAUCC 22-0083) were then amplified and sequenced. Search results showed that the homology of each gene sequence (LSU, ITS, *tub*2 and *rpb*2) of 10 isolates was 100%. And the target sequences of LSU, ITS, *tub*2 and *rpb*2 of four representative isolates were selected. (SICAUCC 22-0080, SICAUCC 22-0081, SICAUCC 22-0082, SICAUCC 22-0083) all sequences are stored in GenBank (Login number: ON707468, ON707463, ON645227, ON645232; ON707469, ON707464, ON645228, ON645233; ON707470, ON707465, ON645229, ON645234; ON707471, ON707466, ON645230, ON645235, respectively). All the population sequences and close species of this genus were selected from this genus, and a total of 73 species were used to construct phylogenetic tree. The sequence contains a combined data set of LSU, ITS, tub2 and rpb2 sequences, which contains 3882 base pairs (bp). Firstly, the maximum-likelihood method is used to analyze phylogeny. Among them, *Leptosphaeria doliolum* and *L.conoidea* were used as external groups for phylogenetic analysis. Phylogenetic tree unites *Nothophoma* genera on the same major clade, four new sequences, SICAUCC 22–0080, SICAUCC 22-0081, SICAUCC 22-0082, SICAUCC 22-0083, are gathered in a well-supported *Nothophoma quercina* (ML = 98%). The results of ML phylogenetic analysis are shown in Fig. 3. Then, the phylogenetic tree is constructed by BI, and the tree shape is the same as ML tree. Isolates SICAUCC 22-0080, SICAUCC 22-0081, SICAUCC 22-0082 and SICAUCC 22–0083 were clustered in this *N. quercina*, which supported the value (ML/BI = 98/1), including *N. quercina* (CBS 633.92) in type species. Four isolates were simultaneously identified as *Nothophoma quercina* (Syd.) Q. Chen & L. Cai. This is the result of the joint identification of phylogenetic trees based on morphological characteristics, cultural characteristics and LSU ITS, *rpb*2 and *tub*2 sequence analysis.

### Pathogenicity tests

**Figure 4:**
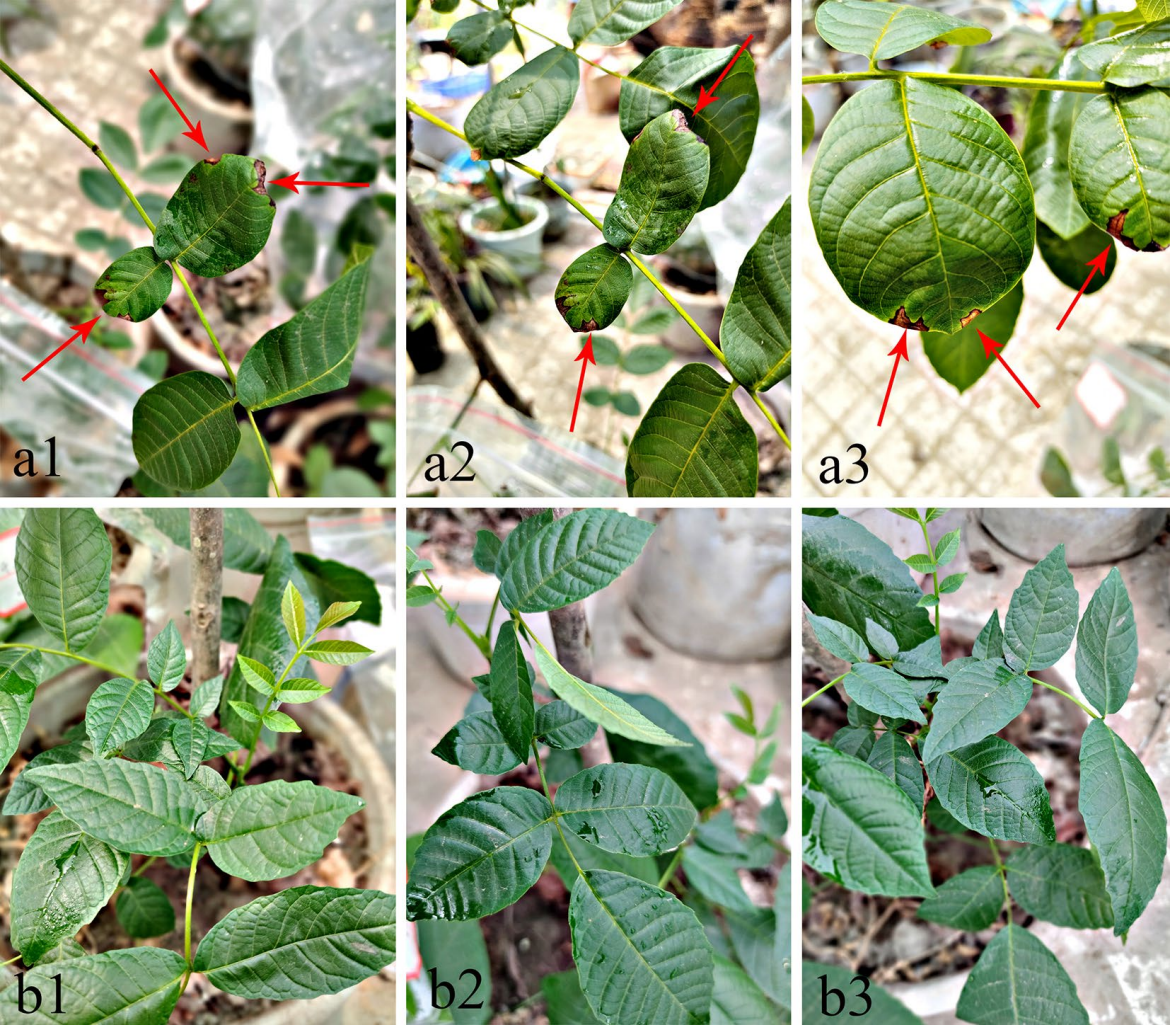
Symptoms on tender leaves in vitro experiments after 15 days inoculated with spore suspension. (**a1**–**a3**) Symptoms of inoculation with spore suspension; (**b1**–**b3**) Symptoms of inoculation with sterile water.

**Figure 5:**
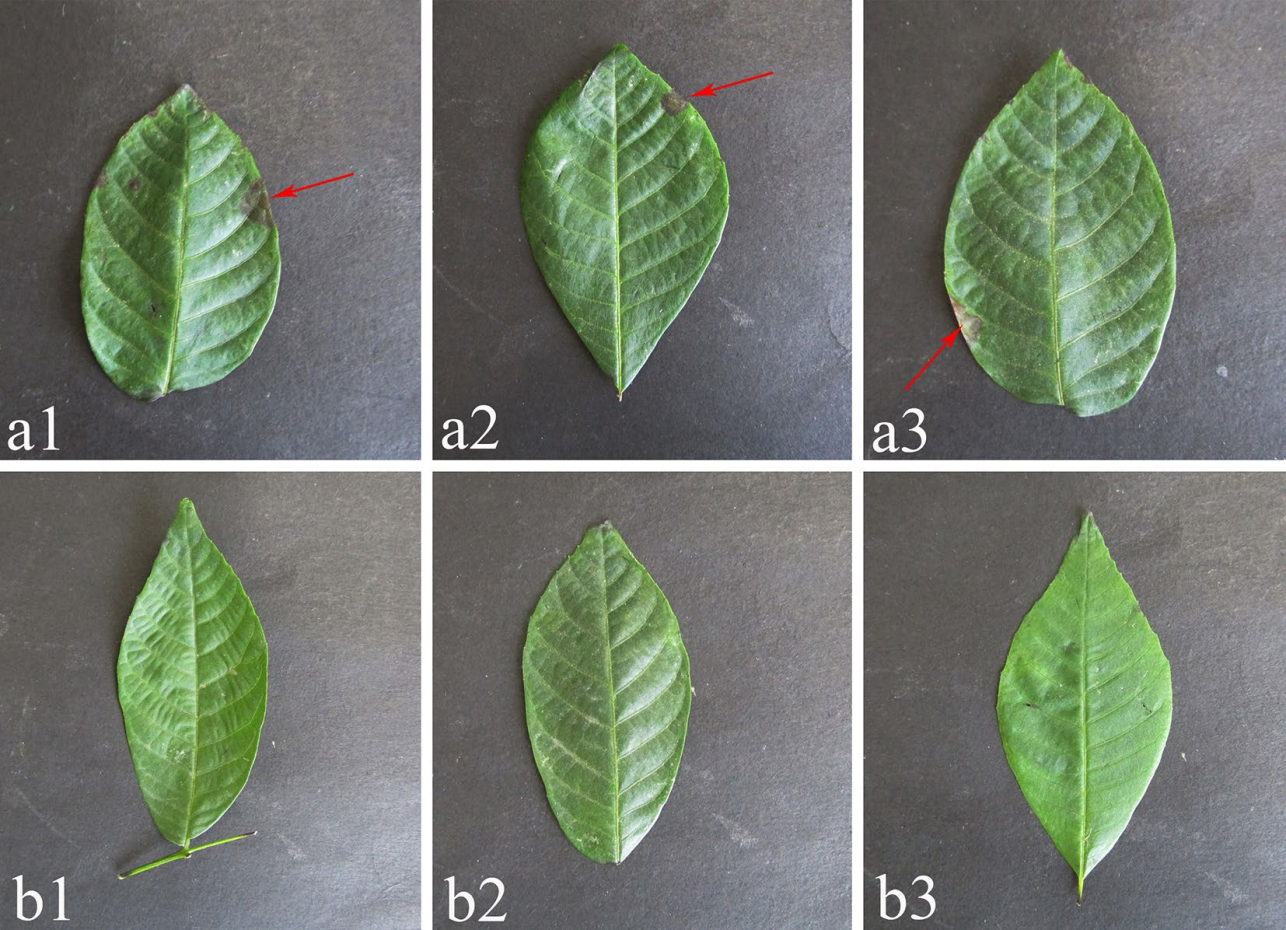
Symptoms observed in vivo experiments after 20 days inoculated with spore suspension. (**a1**–**a3**) Symptoms of inoculation with spore suspension; (**b1**–**b3**) Symptoms of inoculation with sterile water.

The pathogenicity of the tender leaves of living plants was verified in vitro. On the tender leaves that had been detached, conidial suspension (4.5 × 10^5^ conidia/mL) was sprayed onto them. The results showed that the tender leaves in vitro appeared as typical leaf necrosis with light brown to brown spots edge 15 days after inoculation (Fig. 4b1–b3), whereas the control plants were still asymptomatic (Fig. 4a1–a3). After inoculation of healthy walnut seedling leaves for 20 days, the symptoms were the same as those observed in the field (Fig. 5a1–a3), whereas the control plants were still asymptomatic (Fig. 5b1–b3). Fungi were re-isolated from all infected leaves, which were similar to *Nothophoma quercina* by comparison of culture characteristics and morphology. The re-isolated strain was identified as *Nothophoma quercina* by sequencing analysis following Koch’s postulate.

## Discussion

*Nothophoma quercina* is the first confirmed species in this genus, thus confirming the status of *Nothophoma*. *N. quercina* was discovered and named by Chen et al.^2^. This species was originally identified as *Cicinobolus quercinus*, and then transferred to *Ampelomyces* genus. Later, Aveskamp et al.^30^ regarded it as one of *Phoma* and renamed it. Phylogenetic analysis of multiple genes showed that this species gathered in in the *Nothophoma*. Finally, it is proposed that *N. quercina* as a new combination creates *Nothophoma* genus. To date, 22 species have been identified, including *N. anigozanthi, N. arachidis-hypogaeae, N brennandiae, N. chromolaenae, N. eucalyptigena, N. acaciae, N. ferruginea, N. macrospora, N. pruni, N. infossa, N. infuscata, N. multilocularis, N. garlbiwalawarda, N. naiawu, N. nullicana, N. prosopidis, N. pruni, N. spiraeae, N. quercina, N. raii,* and *N. variabilis, N. gossypiicola*^3,4,18,31–37^.

In this study, molecular verification of *Nothophoma quercina* was performed using polygene phylogenetic analysis. For the identification of the pathogen of walnut brown spot caused by *N. quercina* in Sichuan, China, this experiment adopted the combination of morphology and molecule for the first time. The morphological characteristics of the isolated strains are consistent with those described by *N. quercina*. However, due to different nutritional conditions and temperature changes, there are slight differences among different strains. Identification of pathogen of walnut leaf spot is the key of successful control of this disease. Therefore, it is extremely important to correctly identify pathogenic fungi.

*Nothophoma quercina* shoot blight, causes cankers. Among them, there are many reports on leaf diseases of various plants, including Garryaceae, Rhamnaceae, Rosaceae, Rhamnaceae, Anacardiaceae, Oleaceae, Ulmaceae and Fagaceae. *N*. *quercina* is morphologically and geographically diverse. This species has been found in *Aucuba japonica* (China), *Ziziphus jujuba* (China), *Magnolia coco* (China), *Pseudocydonia sinensis* (Korea), *Chaenomeles sinensis* (Korea), and *Prunus dulcis* (Tunisia)^6–8^^,^^38–40^. In the world, we first discovered the brown spot of walnut leaves caused by *N*. *quercina*.

Walnut is an important economic tree species, oil tree species, and ecological tree species worldwide^41^. Walnut is widely planted all over the world, especially in Europe, Asia and many parts of America^42^. Because it is loved by people, it is called one of the four dried fruits, along with cashew nut, hazelnut and almond. The walnut germplasm resources are abundant in China. At present, 13 species of *Juglans* have been identified, with more than 300 cultivated varieties. Among them, *Juglans regia* and *J. sigillata* have the largest planting areas^43^. However, the aggravation of diseases and insect pests is constantly affecting the healthy development of walnut industry. In particular, *Nothophoma quercina* induced leaf diseases lead to early defoliation and fruit loss of *J. regia*. *N. quercina* is a common pathogen in plants. Plant infections are gradually increasing and the scope of damage is constantly expanding. Therefore, it is important to investigate this disease. In this study, the pathogen was identified by morphological and molecular biology methods. Finally, it was verified by pathogenicity test. But these are only the first steps of disease control. At present, further research is being carried out to obtain the mode of occurrence and development of diseases. Understand when and how pathogens infect plants in the growing season, and find out all kinds of environmental factors conducive to disease development. Precise information regarding the disease cycle and epidemiology is required to provide walnut growers with effective management recommendations. Get accurate information about the epidemic of diseases, and provide effective management suggestions for walnut growers according to the information.

## Data Availability

The data presented in this study are deposited in the NCBI GenBank (accession numbers ON707468, ON645232, ON707463, ON645227; ON707469, ON645233, ON707464, ON645228; ON707470, ON645234, ON707465, ON645229; and ON707471, ON645235, ON707466, ON645230.
